# Customized measuring station for Peltier modules^☆^

**DOI:** 10.1016/j.heliyon.2024.e25743

**Published:** 2024-02-07

**Authors:** Roland Binninger, Sabrina Unmüßig, Marc Vergez, Markus Bartel, Olaf Schäfer-Welsen

**Affiliations:** Fraunhofer Institute for Physical Measurement Techniques, Georges-Köhler-Allee 301, Freiburg im Breisgau 79110, Germany

**Keywords:** Peltier module, Measurement system, Characterization, Long-term stability, *COP*

## Abstract

Peltier modules control temperatures in niche products such as wine coolers and camping fridges or are used to precisely temper processes as for example the duplication of DNA sections. Accurate characterization and long term stability of the modules is crucial in order to meet the high reliability requirements especially in critical applications such as in space or medical technology markets. While most of the measurement stations are designed for thermoelectric generators, especially in regard to long term testing, the measurement of peltier modules is rarely described. In this contribution, we show a customized measuring station specifically for the combination of the characterization of peltier modules and their long term stability. With this measurement station, it is possible to determine temperature dependent properties such as the maximum current $I_{\max}$, the maximum cooling power , the maximum temperature difference  and the coefficient of performance (*COP*) while performing cycling tests in between the characterization measurements. In this setup, both sides consist of water driven heat exchangers with a range of  to . The heat flow through the module is measured via two graphite heat flux meters.

Thereby, it is possible to completely characterize a peltier module within this measuring system. An error analysis for the measured properties is given as well. In addition to the characterization of the modules, the long-term stability of the modules can be measured not only with static current and temperature but also at cyclical stimulation of temperature or current changes meaning that application-oriented long-term tests are possible.

## Highlights

• Full automatic characterization of Peltier modules.
• Determination of the characteristic curves and the coefficient of performance.
• Long-term measurements with varying temperatures or varying currents for application-oriented long-term tests.

## Graphical abstract

**Figure:**
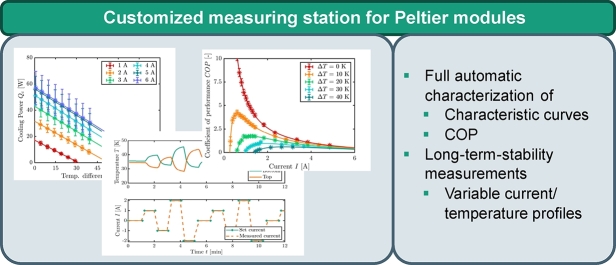

## 1 Introduction

**Figure 1:**
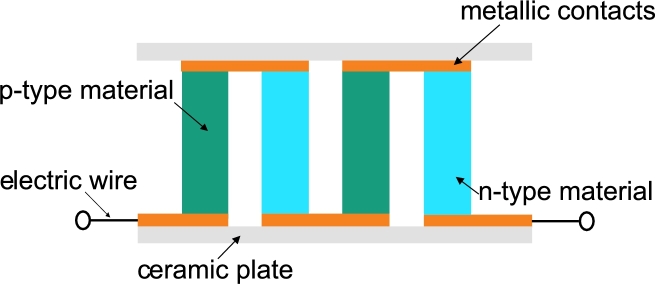
Schematic structure of a typical thermoelectric module. The module consists of *n*- and *p*-type semiconducting material which is connected via metallic contacts. The metallic contacts are in between ceramic plates.

In 1821, Seebeck discovered that a potential difference between the ends of a metal rod arises when a temperature difference is applied [1]. Only thirteen years after the discovery of the so called Seebeck effect, Jean Charles Athanase Peltier found that a current at the junction of two conductors leads to a temperature difference between them [2]. Peltier modules – also known as thermoelectric coolers (TECs) – make use of the Peltier effect for instance to pump heat in several applications. The cooling rate $\overset{˙}{Q}$ of a Peltier module can be described via

(1) $$\overset{˙}{Q}\;=\;(\Pi_{\text{A}}-\Pi_{\text{B}})I\;=\;\Pi_{\text{AB}}I,$$

wherein $\Pi_{i}$ represents the Peltier coefficient of the conductors A and B, $\Pi_{\text{AB}}=\Pi_{\text{A}}-\Pi_{\text{B}}$, and *I* represents the current [3]. In Fig. 1, a schematic drawing of a typical thermoelectric module is shown [3]. One pair of legs consists of a “*p*”- and “*n*”-leg, wherein the letters indicate the type of the semiconducting material. The legs are connected with metallic contacts. Commonly, the metallic contacts are mounted in between ceramic plates. The thermoelectric module is electrically connected in series and thermally connected in parallel. The semiconducting material used in most of room temperature based applications is Bismuth Telluride (Bi_2_Te_3_) [4], with the solid solution alloys Bi_2_Se_3_ (*n*-type) and Sb_2_Te_3_ (*p*-type) [5].

Nevertheless, the usage of thermoelectric modules for cooling or heating is so far only found in niche markets since the majority of cooling technology at room temperature is compressor-based [6]. Some of the advantages in using peltier modules are:

• a large temperature stability
• fast response time
• usability in a broad temperature range
• no moving parts or vibrations
• no use of flowing coolants
• usability with DC-currents

Therefore, typical applications are in sensitive components (such as lasers or CPUs [7], [8]), in medical engineering (PCR-technology [9]) or in wine coolers as well as car seats [10], [11]. Due to the industrial and application-oriented use, it is important to have a broad knowledge about the module and its characteristic properties. This applies not only to the maximum current $I_{\max}$, the maximum cooling power  or the coefficient of performance *COP*, but also to the long-term stability of the modules in different use case scenarios.

The characterization of thermoelectric modules mostly relates on thermoelectric generators (TEGs) [12], [13], [14], [15], [16], [17], [18] or concerns thermoelectric properties such as the Seebeck coefficient or the thermal resp. electrical conductivity [19]. In general, TECs and TEGs have the same set up, although for example the design of the legs (dimensions, cross-sectional area) differs. Yet, the working principle of both – and therefore the characterization properties and quantities – is different. As a consequence, for characterization measurements one is in need of different measurement set ups.

The long term stability of peltier modules is important for the usage in applications like wine coolers or PCR-cyclers. In literature, there already exist studies concerning the long-term stability of thermoelectric generators ([20], [21], [22], [23], [24]) and in thermoelectric coolers ([25], [26]) and moreover the change of material parameters such as the *ZT*-value or the Seebeck coefficient with ageing of the module. In these TEC long-term measurement set ups, often one side is thermally coupled to air via convection. In order for a more instantaneous dissipation of the resulting heat, it is more beneficial to couple both sides to a heat sink and a heat source. Furthermore, neither does literature provide a coupled characterization measurement set up of TEC characteristics and long-term stability, nor the general characterization of peltier modules regarding their *COP*.

In this research study, we show a peltier measuring system that combines a full characterization – namely the measurement of the characteristic curves and COP measurements – of peltier modules including the possibility of long-term tests. In this set up, it is possible to apply variable currents between  and temperature differences between  and , implying that also varying current and/or temperature profiles can be driven for long-term measurements. Both sides of the module are coupled to a recirculating chiller unit. The heat flux meter for the calculation of the cooling power is exchangeable and the atmosphere is arbitrary. Depending on the used TEC, the contact pressure can be selected to suit the module specifications. The measurement process is fully automatic and it is possible to create data sheets based on the measurement results.

In addition, it should be noted that the focus in this research study is a measuring station that combines both the characterization measurements and the long-term measurements. The main advantage of this is that the module no longer needs to be removed and installed between the individual tests and characterizations. Above all, this reduces the error caused by not absolutely identical installation, as the module cannot be clamped in exactly the same manner every time. The authors remark that there are already measuring stations for the characterization of TECs, but a combination of both long-term tests and characterization measurements with an identical setup has not yet been reported. A further advantage in comparison to the long-term measurement set ups for Peltier modules from literature is that both sides of the module are thermally coupled to chiller units so that the heat transport on one of the sides does not occur due to free convection. This makes this measurement site more application-oriented

## 2 Methods

### 2.1 Set up

**Figure 2:**
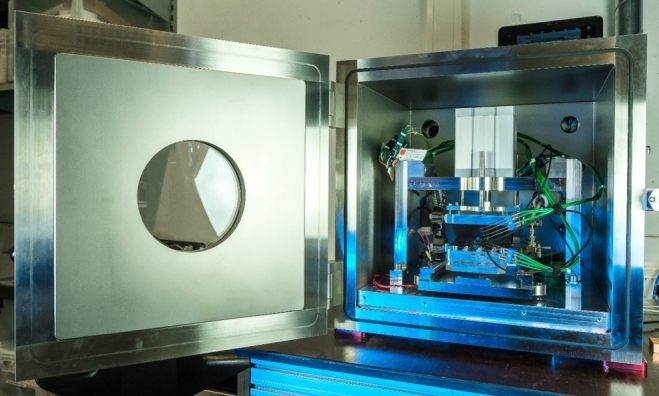
Total view of the measuring system. The vacuum chamber is captured as well. In this set up, peltier modules can be characterized with varying atmospheres (argon, nitrogen, air, vacuum). A more detailed view with a more detailed description of the individual parts can be found in Fig. 3.

**Figure 3:**
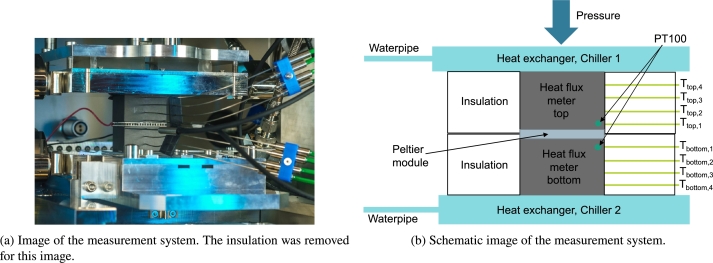
Design of the peltier measuring station. a) Close-up image of the measurement system. b) Schematic image of the measurement system. With this set up, both the characterization measurements and the long-term cycling is possible. The peltier module is clamped between two heat flux meters. The temperature gradient in the heat flux meters is measured with calibrated resistance thermometers (PT100). The inner set up is insulated with a flexible insulation material. The whole set up is placed inside a chamber wherein the atmosphere can be varied.

A total view of the measurement system can be found in Fig. 2. A closer look up of the measurement system and a schematic image of the set up can be found in Fig. 3. The employed devices and measuring equipment are summarized in Table 2 in the Appendix (Section A.1). The set up is placed in a vacuum chamber by Pfeiffer (50 x 50 x 50 cm^3^). The module can be measured under vacuum conditions or under certain defined gas conditions as nitrogen, argon or air. The pressure inside the chamber is adjustable between 0.001 mbar and ambient pressure. The contact pressure is freely adjustable with a pneumatic cylinder (ADN-125-80-I-P-A-30K8-S6). The maximal force is approximately  resulting in a maximum contact pressure of  for standard 40 x 40 mm^2^ modules. As can be seen in Fig. 3b the cold and the hot side are coupled via two recirculating chiller units (on the cold side: Huber Unichiller 015w-G, on the hot side: Lauda VarioCool VC 1200). Both chillers are filled with a water-glycole mixture and can be used between  and . Two heat flux meters (HFM) are placed above and below the module. During this research study, we used graphite but for module measurements with smaller cooling powers, it is also possible to implement (stainless) steel or aluminum HFMs. The cross-sectional area of the HFM corresponds to the cross-sectional area of the characterized module whereas the height is chosen so that a sufficiently large temperature gradient can be measured within the HFM. Four resistance thermometers (PT100) are used to measure the temperature gradient in each HFM. A schematic of the design can be found in the appendix (see Fig. 14 in the Appendix in Section A.2 for the exact dimensions and the spacing between the PT100s). To set a temperature difference at the peltier module, the chillers are controlled via another PT100 inside the HFM. The distance between these PT100 and the module is . The peltier module can be of rectangular (between 10 x 10 and 100 x 100 mm^2^) or circular shape (between  and  in diameter). For the experiments shown here, a peltier module by European Thermodynamics with a cross sectional area of 40 x 40 mm^2^ (ET-127-14-15; for more information, see [27]) was used.

### 2.2 Determination of the cooling power and error propagation

The heat flux $\overset{˙}{Q}$ inside of the heat flux meter is calculated via Fourier's law

(2) $$\overset{˙}{Q}=kA\frac{\Delta T_{\mathrm{HFM}}}{\Delta x}.$$

**Figure 4:**
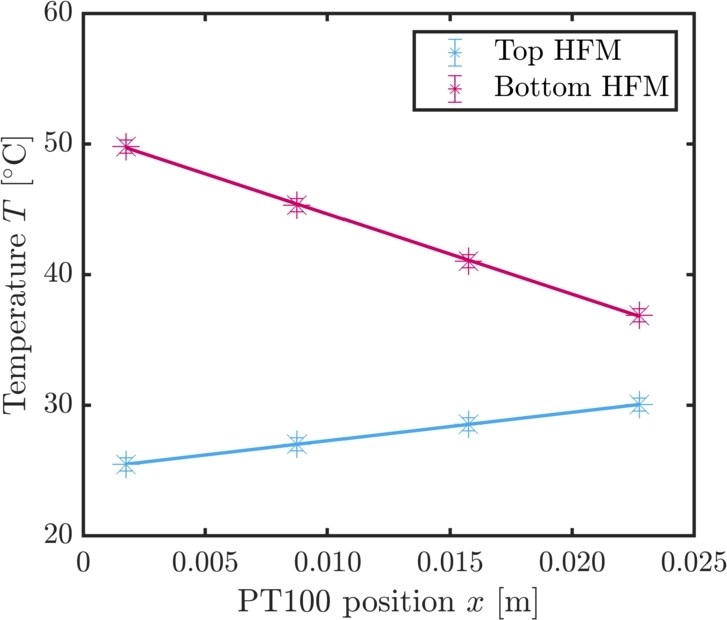
Temperature gradient in the graphite block for the top and bottom heat flux meter. Here, the temperature difference between the hot and the cold side is  at a current of 4 A. The solid lines represent a linear regression based on the measurement data. The result of the linear model is used for the calculation of the heat flux and therefore for the calculation of the cooling power.

**Figure 5:**
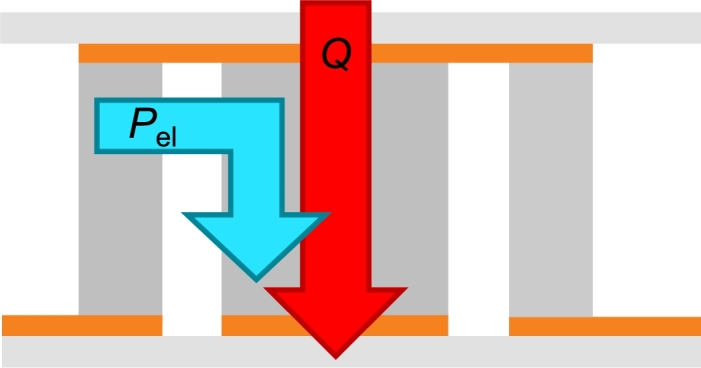
Illustration of the heat flux in the peltier module. In our here shown measurements, the heat flows from the top to the bottom. The dissipated heat resulting from the electrical power reduces the total cooling power of the module by *P*_el_.

**Figure 6:**
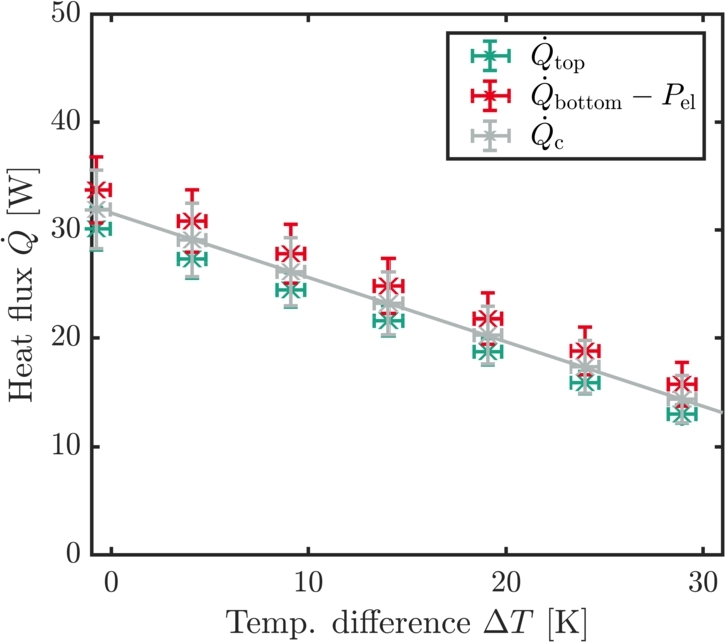
Cooling power versus temperature difference at . One single data points consists of the mean value of the heat flux for one temperature difference as described in the text. The electrical power is subtracted from the heat flux of the bottom HFM. For the linear fit as depicted by the solid line, the mean value is used.

*k* stands for the thermal conductivity of the heat flux meter, *A* is the cross-sectional area of the HFM and $\Delta T_{\mathrm{HFM}}/\Delta x$ is the temperature gradient over the spatial difference of the PT100 inside the heat flux meter. During the data analysis, for the graphite HFM a thermal conductivity of  is used. For the calculation of the thermal conductivity, see Section A.3 in the Appendix. The cross-sectional area is dependent on the size of the peltier module. The heat flux meter matches this area as well and for the error calculation it is assumed that the uncertainty of the cross-sectional area is negligible in comparison to the other uncertainties. In Fig. 4, the temperature gradient in both heat flux meters is drawn against the position of the PT100 *x* as indicated by Fig. 3b. During this measurement, a temperature difference of  was applied at the peltier module. The *y*-error bars correspond to the uncertainty of the PT100 and are namely . The slope $\Delta T_{\mathrm{HFM}}/\Delta x$ is determined via a weighted linear regression with the model equation

(3) $$T\;=\;\frac{\Delta T_{\mathrm{HFM}}}{\Delta x}x+T_{0},$$

wherein $T_{0}$ is the *y*-intercept. The cooling power $Q_{c}$ of the peltier module corresponds to the heat flux inside of the heat flux meter as defined in Eq. (2). Since in our example, the heat flows from the top of the peltier module to the bottom, the top heat flow meter shows an increase in temperature and the bottom heat flow meter shows a decrease in temperature. The dissipated heat that results from the electrical power reduces the cooling power of the module by $P_{\mathrm{el}}=U\cdot I$, leading to a total cooling power of

(4) $$Q_{c}=\frac{\left|{\overset{˙}{Q}}_{\mathrm{top}}|+(|{\overset{˙}{Q}}_{\mathrm{bottom}}|-P_{\mathrm{el}}\right)}{2},$$

as also illustrated in Fig. 5. $\left|{\overset{˙}{Q}}_{\mathrm{top}}\right|$ and $\left|{\overset{˙}{Q}}_{\mathrm{bottom}}\right|$ represent the absolute heat flux inside of the top resp. the bottom heat flux meter. The voltage *U* is directly transmitted to the Keithley multi meter whereas the current *I* has to be determined via a shunt resistance. As will be described in Section 2.3, the measurements for the determination of the cooling power are repeated sufficiently which means that the depicted cooling powers are at all times mean values. An example can be seen in Fig. 6. Since the measurements are multiple measurings, the error on the quantities comprises of a statistical and a systematical part. The statistical error $s_{i,\mathrm{stat}}$ on the physical quantity *i* is calculated via

(5) $$s_{i,\mathrm{stat}}\;=\;\frac{\sigma_{i}}{\sqrt{N}},$$

wherein $\sigma_{i}$ is the standard deviation of said quantity and *N* is the number of measurements. The total error is then calculated with

(6) $$s_{i,\mathrm{tot}}\;=\;\sqrt{s_{i,\mathrm{stat}}^{2}+s_{i,\mathrm{sys}}^{2}},$$

wherein $s_{i,\mathrm{sys}}$ represents the systematic uncertainty. Collectively, the total errors for the electrical quantities and the temperature can be summarized to

(7) $$s_{U}\;=\;\sqrt{s_{U,\mathrm{stat}}^{2}+{\left(0.02\text{\%}U\right)}^{2}},$$

(8) $$s_{I}\;=\;\sqrt{s_{I,\mathrm{stat}}^{2}+{\left(0.02\text{\%}I\right)}^{2}},$$

(9)
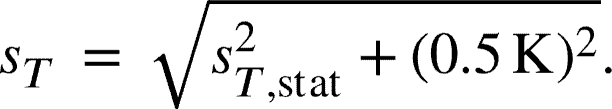
 For the electrical power and the cooling power, the relative Gaussian error propagation is used, resulting in

(10) $${\left(\frac{s_{P_{\mathrm{el}}}}{P_{\mathrm{el}}}\right)}^{2}\;=\;{\left(\frac{s_{U}}{U}\right)}^{2}+{\left(\frac{s_{I}}{I}\right)}^{2},$$

(11) $${\left(\frac{s_{{\overset{˙}{Q}}_{\mathrm{top}/\mathrm{bottom}}}}{{\overset{˙}{Q}}_{\mathrm{top}/\mathrm{bottom}}}\right)}^{2}\;=\;{\left(\frac{s_{k}}{k}\right)}^{2}+{\left(\frac{s_{b}}{b}\right)}^{2}.$$

In the latter case, $b=\Delta T_{\mathrm{HFM}}/\Delta x$ represents the slope of the linear regression with the error $s_{b}$ from the linear fit

(12) $$s_{b}=\sqrt{\frac{\sum_{i}w_{i}}{\delta }\sigma_{T}^{2}},$$

with

(13) $$\delta \;=\;\sum_{i}w_{i}\sum_{i}w_{i}x_{i}^{2}-{\left(\sum_{i}w_{i}x_{i}\right)}^{2}$$

and the weighting $w_{i}=1/s_{T_{i}}^{2}$.

Finally, the error on the cooling power is

(14) $$s_{Q_{c}}\;=\;\sqrt{s_{{\overset{˙}{Q}}_{\mathrm{top}}}^{2}+s_{{\overset{˙}{Q}}_{\mathrm{bottom}}}^{2}+s_{P_{\mathrm{el}}}^{2}}.$$

### 2.3 Characterization measurements

With the shown measuring system, it is possible to characterize the TEC in a variety of quantities. In the following, all characterization possibilities are shown and shortly explained. In the case of the maximum current measurement, a flow chart is shown to illustrate the software routine. The routines for all the characterization measurements are implemented via LabVIEW.

#### 2.3.1 Determination of $I_{\max}$

**Figure 7:**
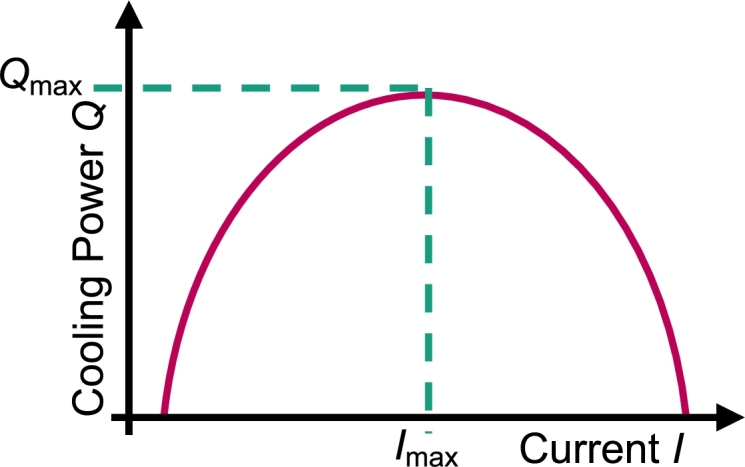
Schematic drawing of the maximum current of the peltier module. Both the top and the bottom side of the peltier module are held at the same temperature.

**Figure 8:**
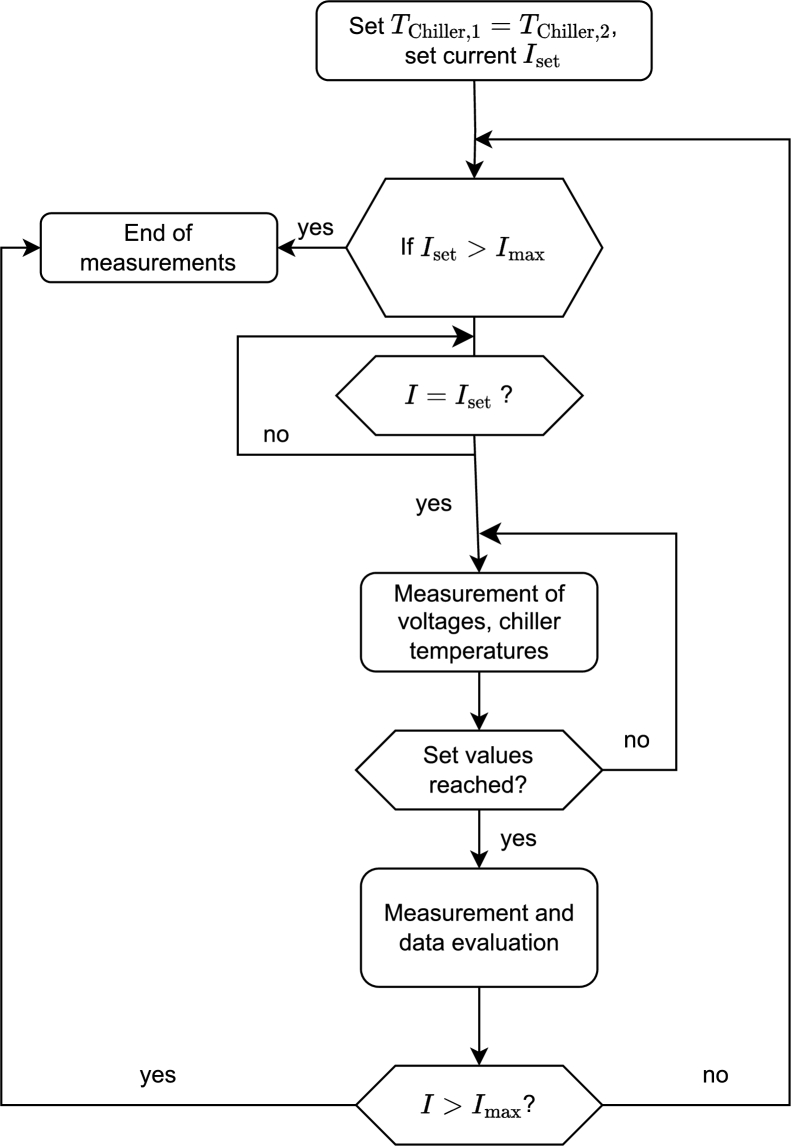
Scheme of the measurement routine for determination of the maximum current of the peltier module.

In principle, the cooling power and the resulting temperature difference of a TEC is adjusted by the current. If the cooling power is measured in dependence of the current when the top and the bottom side of the module are held at constant temperature, the cooling power increases up until a maximum current $I_{\max}$. For larger currents, the cooling power decreases. Therefore, $I_{\max}$ corresponds to the maximum cooling power  at no temperature difference, as can be seen in Fig. 7. Fig. 8 shows the LabVIEW routine for the $I_{\max}$ measurement. Before the measurement starts, an initial set current and a current step size have to be selected. The increment determines the increase in the current after each measurement sequence. The temperature of the hot and the cold side of the peltier module is adjusted via the PT100s that regulate the chiller temperatures to the chosen temperature. If the temperature of the peltier module does not change over a sufficient amount of time, the temperature gradient in the heat flux meters is determined in a multiple measuring so that the cooling power can be calculated as shown in Section 2.2. The LabVIEW routine internally calculates the cooling power at a given current. Then, the current increases by the step size and the measurement is repeated. The cooling power is compared with the previous result. If the cooling power decreases, the measurement routine stops and the measurement series is evaluated. With the LabVIEW program, it is possible to model the cooling power in dependence of the current with a parabolic fit ($f(I)=aI^{2}+bI+c$) as well. The error on the maximum current can be calculated with Eq. (8), whereas the error on the cooling power can be calculated with Eq. (14).

#### 2.3.2 Determination of the characteristic curves

**Figure 9:**
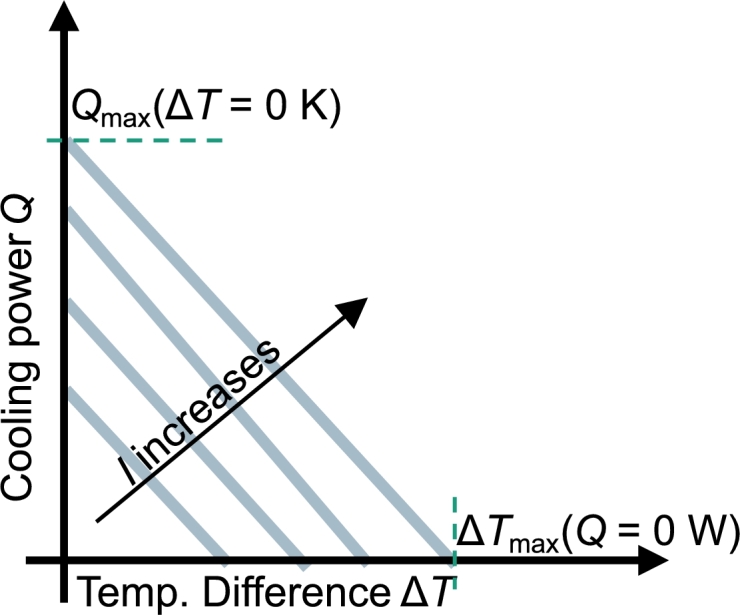
Schematic drawing of the measurement of the characteristic curves of the peltier module. With an increasing current (up to *I*_max_), the cooling power and the temperature difference increases. Before the measurement, the step size for the current and for the temperature differences at a given current can be freely chosen.

For the measurement of the characteristic curves, it is useful to obtain the value for $I_{\max}$ beforehand since – as explained before – the peltier module oversteers at larger currents which makes it unnecessary to measure larger currents. In the LabVIEW interface, it is possible to choose the number and the values of the measured currents. Therefore, it is also possible to only measure the $I_{\max}$-curve to determine the maximum possible cooling power  and the maximum temperature difference . A scheme of characteristic curves and their characteristic values can be found in Fig. 9. The temperature differences at which the cooling power is measured have to be selected as well. Usually, the hot side temperature is held at ,  or . Therefore, only the cold side temperature changes after one measurement series is completed. The characteristic curves are measured current by current. If the current is steady, the temperature difference of the peltier module is adjusted by the chillers. If the temperatures do not change over a certain period of time as given by a selectable accuracy the cooling power of the module is measured via a temperature decrease in a multiple measuring. After the heat flow is measured, the program automatically adjusts for the next current. For the determination of the cooling power and the associated error, see Section 2.2. To obtain the characteristic curves, a linear model(15)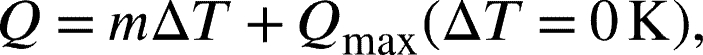 wherein *m* is the slope of the curve, is fitted to the measurement data. The linear regression is weighted with the squared inverse of the errors on the cooling power $1/s_{Q_{i}}^{2}$. The temperature difference at the peltier module is calculated with the nearest PT100s (see Fig. 3b)

(16) $$\Delta T=T_{\mathrm{bottom},1}-T_{\mathrm{top},1}.$$

The error on the temperature difference is(17)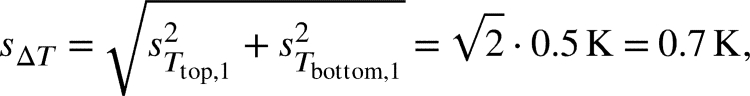 since the error of the temperature of each PT100 is . The *y*-intercept of the regression model  corresponds to the maximum cooling power for a given current when no temperature difference is applied to the peltier module. The *x*-intercept(18)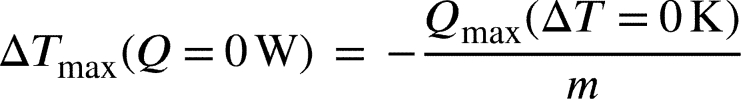 corresponds to the maximum possible temperature difference when no heat is transported through the module. The error on the *y*-intercept is represented by the statistical error(19)
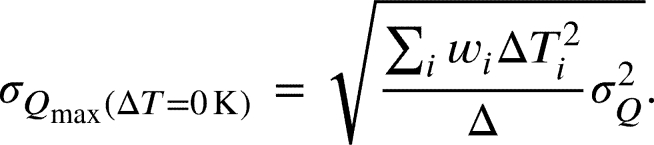
 Here, $\sigma_{Q}$ is the standard deviation of the cooling power and *δ* is

(20) $$\delta \;=\;\sum_{i}w_{i}\sum_{i}w_{i}\Delta T_{i}^{2}-{\left(\sum_{i}w_{i}\Delta T_{i}\right)}^{2}$$

wherein $w_{i}=1/s_{Q_{i}}^{2}$ is the weighting of the measurement data. The error on the *x*-intercept is calculated via(21)
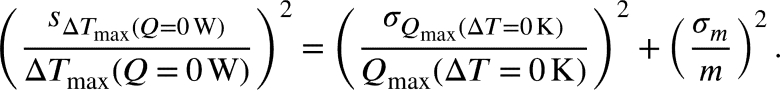

#### 2.3.3 Determination of the *COP*

The coefficient of performance is determined via the ratio of the cooling power versus the electrical power $P_{\mathrm{el}}$

(22) $$COP=\frac{Q_{c}}{P_{\mathrm{el}}}=\frac{({\overset{˙}{Q}}_{\mathrm{top}}+({\overset{˙}{Q}}_{\mathrm{bottom}}-P_{\mathrm{el}}))/2}{P_{\mathrm{el}}}.$$

**Figure 10:**
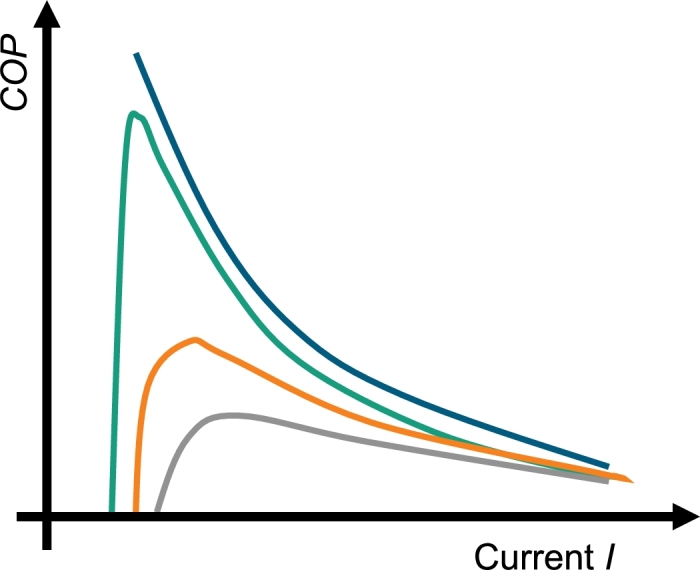
Schematic drawing of a *COP*-measurement for four different temperature differences.

In contradiction to the other measurement routines, here the current is varied at a constant temperature difference at the peltier module. Before the measurement starts, the temperature differences as well as the step sizes of the current have to be chosen. When the current at the module is steady, the temperature difference is regulated via the chiller units. The duration of the temperature regulation can be customized via the accuracy regulation of the temperature at the chillers. If the temperature is steady, the temperature gradient in the HFM is measured and the cooling power is determined. Then, the current is increased and the routine starts again. It has to be noted that for small currents and for small cooling power, it is useful to take a small step size since the *COP* is increasing and decreasing at a fast rate. This can be seen in Fig. 10 where a schematic drawing of the *COP*-measurement is shown for different temperature differences. The error of the *COP* is determined via Gaussian error propagation

(23) $$s_{COP}\;=\;{\left({\left[\frac{s_{{\overset{˙}{Q}}_{\mathrm{bottom}}}}{2P_{\mathrm{el}}}\right]}^{2}+{\left[\frac{s_{{\overset{˙}{Q}}_{\mathrm{top}}}}{2P_{\mathrm{el}}}\right]}^{2}+{\left[\frac{s_{P_{\mathrm{el}}}({\overset{˙}{Q}}_{\mathrm{top}}+{\overset{˙}{Q}}_{\mathrm{bottom}})}{2P_{\mathrm{el}}^{2}}\right]}^{2}\right)}^{1/2}.$$

The uncertainties for the electrical power and the cooling power are found in Eq. (10) and Eq. (11). In order to create a data sheet with the measurement data, a rational model of second order

(24) $$f(I)=\frac{\left(a_{1}I^{2}+a_{2}I+a_{3}\right)}{\left(b_{1}I^{2}+b_{2}I\right)}$$

is fitted to the data. The model equation is based on the formula for the calculation of the *COP* with material properties [3]

(25) $$COP_{\mathrm{mat}}\;=\;\frac{\alpha IT_{c}-\frac{1}{2}RI^{2}-k(T_{h}-T_{c})}{\alpha I(T_{h}-T_{c})+RI^{2}},$$

wherein *α* is the Seebeck coefficient, *R* is the inner resistance of the module, and $T_{i}$ represents the temperature of the hot resp. the cold side.

#### 2.3.4 Long-term measurements

**Figure 11:**
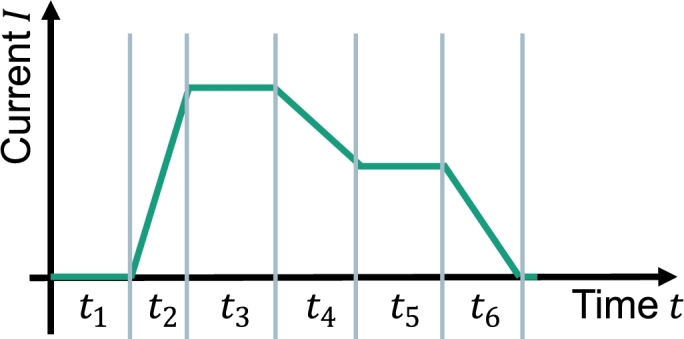
Example of a current profile. To get a whole cycle, different time intervals with varying currents have to be chosen. The number of cycles can be chosen freely before the long-term cycling starts.

Before the cycling starts, the profile and the number of cycles have to be defined. For the time being, it is only possible to regulate the current and set chiller temperatures to a temperature depending on the application scenario. Nevertheless, a temperature difference between the hot and the cold side of the TEC can be applied to be held constant during the cycling. During the long-term cycling, the temperatures at the peltier module are measured as well. A profile is defined via a time interval in which a current or a temperature difference is applied via a ramp or held constant. To illustrate the possibilities, Fig. 11 shows a possible application-oriented current profile in dependence of the time. The cycle automatically starts from the beginning up until the number of total cycles that was defined beforehand is reached.

## 3 Results & discussion

### 3.1 $I_{\max}$-measurement

**Figure 12:**
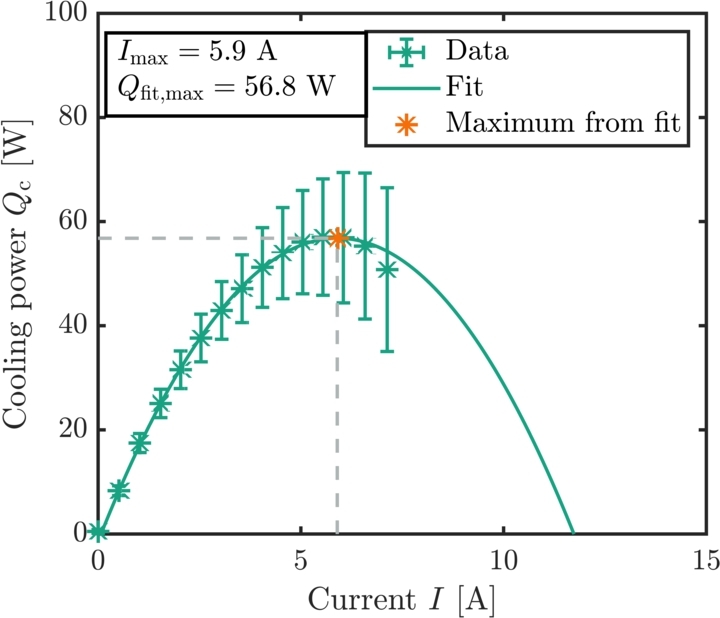
Cooling power versus current. A second order polynomial fit (solid line) is used to model the data. The maximum value for both the current and the cooling power is calculated with the model equation and displayed in the plot by an orange dot. The error bars for the current are not visible.

Fig. 12 shows the cooling power plotted against the current. The temperature on the hot side was held at . The green dots represent the data points and the solid line represents a polynomial fit of second order. It can be seen that the fit corresponds extremely well to the data. The maximum current is found to be  and the maximum cooling power is . The maximum cooling power is determined by the maximum of the fit. The maximum current corresponds to said maximum cooling power.

### 3.2 Measurement of the characteristic curves

**Figure 13:**
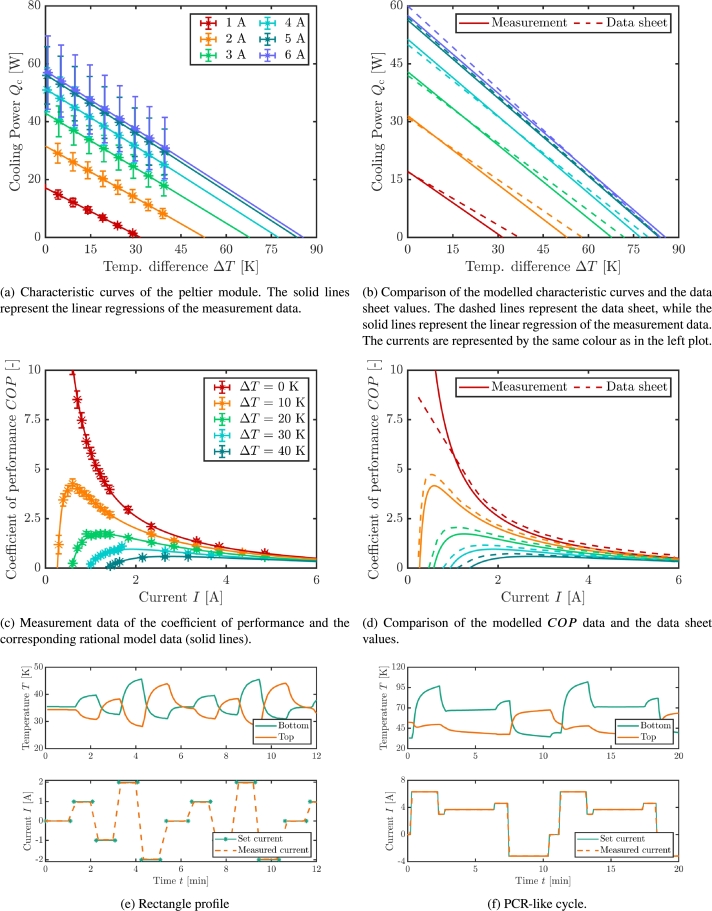
a), b): Summary of the measurement of the characteristic curves. The error bars on the temperature difference are not visible. c), d): Summary of the *COP* measurements The error bars on the current are not visible. e), f): Two examples for long-term measurement profiles. The bottom graphs show the current profile, the top graphs show the temperature profile for the hot and the cold side of the peltier module. The values from the data sheet are taken from [27].

**Figure 14:**
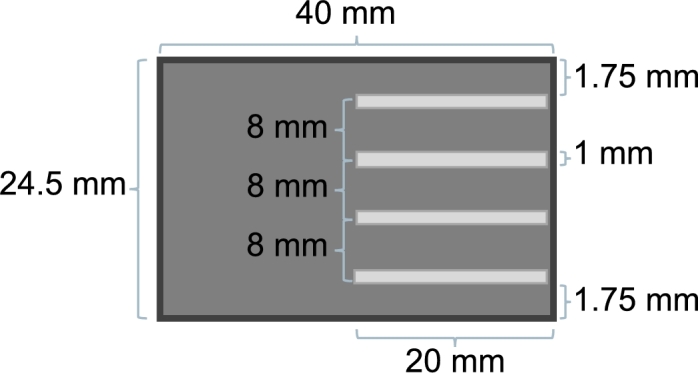
Dimensions of the heat flow meter in the *xz*-axis. The height of the heat flux meter is . The cross-sectional area of the heat flux meter is dependent on the dimensions of the peltier module. During the measurements performed in this study, we used a peltier module of the dimensions 40 x 40 mm^2^. The light shaded grey rectangles indicate the openings for the PT100s. Each has a diameter of 1 mm.

Fig. 13a and Fig. 13b show the summary of the characteristic curve measurement of the peltier module between  and . Fig. 13a shows the measurement data with the corresponding linear regressions (solid lines). Fig. 13b compares the linear regressions with the data sheet lines as found in [27]. Temperature differences between  and  were measured in  steps. Larger temperature differences could not be applied at the module since the temperature inside the recirculating unit would have been out of the specifications. The linear regressions fit the measurement data well. The values for  and  can be found in Table 1. In comparison to the data sheet, the data shows a slight variation at small cooling powers resp. large temperature differences at small currents. One reason for this can be that the measurements were only performed in a range up to a temperature difference of  meaning that deviations in the cooling power for larger temperature spans are not accounted for in the linear regression. Another possible reason is the difficulty of measuring small cooling powers. With a small cooling power comes a small temperature gradient in the HFM and the recirculating chiller units have to be very precise regarding the temperature stability so that the temperature gradient in the HFM can be seen. Nevertheless, the measurements fit the data sheet values very well for small temperature differences and for larger currents (see the 4 to 6 A measurements). On another note, it has to be said, that often the data sheet values as given by the manufacturer are values that are calculated from material properties and not measurements. With this in mind, the shown measurements are in accordance with the data sheet values. A general improvement that holds for the these measurements but also for the $I_{\max}$- and the *COP*-measurement would be an even larger insulation and performing the measurements in vacuum conditions. The here shown measurements are in ambient conditions. This leads to an underestimation of the cooling power due to convection.

**Table 1:** Results for the maximum cooling power and the maximum temperature difference between 1 and .

I [A]	[W]	[K]
1	17.1 ± 0.5	32 ± 6
2	31.57 ± 0.03	52.9 ± 0.2
3	42.94 ± 0.05	67.8 ± 0.2
4	51.37 ± 0.03	77.34 ± 0.09
5	56.33 ± 0.05	83.6 ± 0.2
6	57.56 ± 0.07	85.7 ± 0.2

### 3.3 *COP*-measurement

Fig. 13c and Fig. 13d depict the coefficient of performance versus the current at several temperature differences. Fig. 13c shows the measurement data and the corresponding rational equation model. Fig. 13d compares the model values with the data sheet values as found in [27]. In comparison to the data sheet, the model data overestimates the *COP* at larger currents. Nevertheless, the modelled data is in accordance with the values from the manufacturer.

### 3.4 Long term measurements

Fig. 13e and Fig. 13f show two driven current profiles for possible long-term measurements. As can be seen, the bottom graph of Fig. 13e shows a typical rectangular profile with different maximum currents. Each time interval is  long. The green line shows the current that was defined beforehand in the LabVIEW program. The measured current values follow the set current extremely well. The top graph of Fig. 13e shows the temperature profile of the peltier module for the bottom and the top side. The temperature of the peltier module follows the driving current. The less steep ramp of the measured current (dashed orange line) comes from a delay in the DAQ. The DAQ sets the current and the voltage. At the same time, it is not possible to take measurements with the DAQ leading to a delay in the current measurement. The flat ramp disappears if the current is measured via an oscilloscope. Fig. 13f shows a cycle similar to a PCR-cycle. The measured currents (bottom graph) agree extremely well with the set currents. The temperature of the bottom and the top side of the peltier module is shown in the top graph.

## 4 Conclusion

During this research study, we developed a fully automatic peltier measuring station wherein a thermoelectric cooler can be characterized and the long-term stability of the module can be tested. The characterization measurements contain the maximum current, the characteristic curves as well as the coefficient of performance. Furthermore, a detailed error calculation was carried out. The measured data was compared with the data sheet as given by the manufacturer and shows an excellent agreement. In addition, we showed the application of two different current-time profiles, namely an application oriented PCR-like-cycle and a rectangle profile with a fast change of currents.

In comparison to measurement set ups from literature, both characterization measurements and long-term cycling can be performed at the same measurement station. This was not reported before. Besides, it is also possible to measure the peltier module's coefficient of performance with this set up. For the long-term cycling, it is not only possible to drive current profiles, it is also possible to vary the temperature difference of the recirculating chiller units. In general, all kinds of (application-oriented) profiles are imaginable.

In future work, long-term cycling tests are planned with characterization measurements in between. After reaching a fixed number of cycles, the characteristic curves are to be measured which makes it possible to determine the ageing of the modules by comparison of values such as $I_{\max}$,  or  before and after a given number of cycles. Moreover, it is also possible to model physical properties of the module based on the characterization measurements as for example the thermal resp. electric conductivity and the Seebeck coefficient. By modelling these quantities, the temperature dependent figure of merit *ZT* can be determined.

## CRediT authorship contribution statement

**Roland Binninger:** Writing – review & editing, Formal analysis, Conceptualization. **Sabrina Unmüßig:** Writing – original draft, Visualization, Project administration, Formal analysis. **Marc Vergez:** Validation, Investigation. **Markus Bartel:** Software. **Olaf Schäfer-Welsen:** Writing – review & editing, Supervision, Funding acquisition.

## Declaration of Competing Interest

The authors declare that they have no known competing financial interests or personal relationships that could have appeared to influence the work reported in this paper.

## Figures and Tables

**Table 2:** Summary of the measuring technology.

sensor	type	measurand	range	precision
**Table:** multi meter & multiplex	Keithley 2700/7700	**Table:** voltage, temperature	**Table:** – – (PT100)	**Table:** (DC-V)
power supply	EA-LOE PS-8032-20	current, voltage	,	
resistance thermometer	PT100	temperature	–	
chiller 1	Huber Unichiller 015w-H	temperature	–	
chiller 2	Lauda VarioCool VC1200	temperature	–	

**Figure 15:**
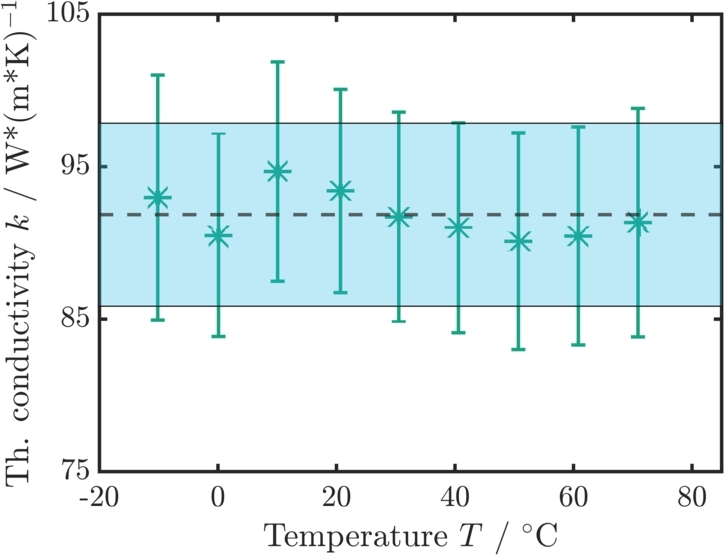
Thermal conductivity data of graphite. The mean value is indicated by a horizontal dotted line. For the determination of the mean value, the data in the relevant temperature interval is used.

## Appendix A

### A.1 Measuring technology

Table 2 contains the used measuring technology that was used during the experiments.

### A.2 Dimensions of the heat flux meter

Fig. 14 shows the dimensions of the graphite heat flux meter and the distances between the notches for the PT100. The cross-sectional area of the heat flux meter is dependent on the dimensions of the peltier module. During the measurements performed in this study, we used a peltier module of the dimensions 40 x 40 mm^2^. The light shaded grey rectangles indicate the openings for the PT100s.

### A.3 Thermal conductivity measurement of graphite

The thermal conductivity of the heat flux meter is needed for the calculation of the cooling power of the thermoelectric module. One possibility to obtain the thermal conductivity is via the laser flash analysis (LFA) [28]. The used analyser is by Netzsch (LFA 457 MicroFlash). The thermal diffusivity $t_{d}$ (accuracy  according to the manufacturer) and the specific heat capacity $c_{p}$ (accuracy  according to the manufacturer) were measured. With the density of the sample *ρ*, the thermal conductivity *k* can be expressed via

(26) $$k(T)=\rho t_{d}(T)c_{p}(T).$$

For each temperature that was measured, five measurements of the thermal diffusivity and three measurements for the specific heat capacity were performed. At each temperature, the mean value was taken for both quantities. Then, the thermal conductivity is calculated for that temperature. The density of the graphite material is $\rho =1.81\text{ kg}/{\text{m}}^{3}$ and is assumed to be constant over the measured temperature range. Fig. 15 shows the thermal conductivity of graphite in the interval between  and . The dotted line indicates the mean value of the thermal conductivity over the whole temperature range  that is calculated by taking the mean value for the thermal conductivities over the shown temperatures. The filled blue area indicates the error of the thermal conductivity as described below. The error of the mean value comprises of a statistical uncertainty and a systematical error. The statistical uncertainty $s_{k,\mathrm{stat}}$ is calculated via Eq. (5). The systematic error $s_{k,\mathrm{sys}}$ is calculated via the relative Gaussian error propagation

(27) $${\left(\frac{s_{k,\mathrm{sys}}}{k}\right)}^{2}={\left(\frac{s_{c_{p}}}{c_{p}}\right)}^{2}+{\left(\frac{s_{t_{d}}}{t_{d}}\right)}^{2}$$

with the accuracy as given by the manufacturer. In comparison to the uncertainties for $c_{p}$ and $t_{d}$, the uncertainty for the density can be neglected. The total error is then given by Eq. (6).
